# The adoption non-adoption dichotomy: Why do smallholder producers dis-adopt improved chicken breeds?

**DOI:** 10.1371/journal.pone.0310060

**Published:** 2024-10-31

**Authors:** Mulugeta Y. Birhanu, Girma T. Kassie, Tadelle Dessie

**Affiliations:** 1 International Livestock Research Institute (ILRI), Addis Ababa, Ethiopia; 2 Social, Economics, and Policy Research Team, International Centre for Agricultural Research in the Dry Areas (ICARDA), Addis Ababa, Ethiopia; Colorado State University, UNITED STATES OF AMERICA

## Abstract

Adopting agricultural technologies is crucial to improve productivity and livelihoods in developing countries. While much research has focused on adoption decisions, understanding dis-adoption, when farmers stop using technology, is equally important. Studies on agricultural technology adoption often treat dis-adopters (those who initially adopted but later discontinued to use) and never-adopters (those who never adopted) as the same, using binary models to analyze farmers’ decisions. We argue that a better understanding of these decisions can be achieved by separately analyzing ’never-adoption’, ’dis-adoption’, and ’adoption.’ Using nationally representative data from three African countries, Ethiopia, Nigeria, and Tanzania, we developed a multinomial logit model to analyze the adoption of improved chicken breeds. Our findings show that dis-adopters of improved chicken are different from never-adopters. Factors associated with dis-adoption include gender and education of household heads, access to training and extension services, breeding and culling practices, access to markets, use of complementary inputs, production objectives, landholding size, income diversity, and access to finance. Policies and strategies that aim to enhance sustained adoption and use of improved chicken breeds should promote a bundle of technologies, including tailored training, women empowerment, locally adapted and farmer-preferred chicken breeds, complementary inputs and services, innovative marketing strategies, and delivery models for bundles of technologies.

## 1. Introduction

Smallholder chicken production is crucial for the livelihoods of rural and peri-urban households in developing countries [1–6]. Despite its significant role in sub-Saharan Africa, the sector’s production and productivity remain low due to the limited egg and meat productivity of current breeds and the prevalent low-input, low-output traditional production system [7]. Previous efforts to improve the sector include the introduction of high-yielding exotic breeds, improving smallholder management practices, and enhancing the genetic potential of existing species through selection and crossbreeding. However, the adoption and sustained use of technology have remained very low, with considerable rates of dis-adoption[8,9]. Limited empirical research indicates that low adoption of improved chicken is associated with household socioeconomics, farm characteristics, as well as policy and institutional factors [10–12].

Studies on the adoption of improved chicken typically frame adoption as a dichotomy—either adopting or not adopting—with the latter category including both dis-adoption and never-adoption. This approach has led to limited comparative evidence on adoption and dis-adoption decisions. However, farmers who have tried and stopped using technology are not necessarily the same as those who have never tried it. These two groups may have different experiences, perceptions, and understanding of the technologies that can influence their decisions to use them in the future. Therefore, separating these groups can offer additional insight into understanding the dynamics of improved chicken adoption decisions and the sustained use of technologies, leading to distinctive policy implications.

Empirical dis-adoption studies historically focused on technologies related to crops and natural resources [12–20]. These studies revealed that the decisions to dis-adopt farm households are related to the lack of continuous technical support, the inability of technologies to meet the expectations of farmers, the profitability of technologies, the distance to markets or services providers, group memberships, age and sex of farmers, literacy, access to input and extension services, labor intensiveness of technology, and consumer preference.

Studies on improved poultry breed adoption in general and improved chicken breed dis-adoption more specifically are very limited. Therefore, using national-level data and context-specific indicators across Ethiopia, Nigeria, and Tanzania, we explore factors associated with the adoption status of improved chicken. We estimated a multinomial logit model to examine the relative importance of the factors influencing smallholder chicken producers’ decisions to ‘adopt’, ‘dis-adopt’, and ‘never-adopt’. This study adds insights to the existing body of knowledge in three ways. First, we used national-level data from three countries and presented evidence from a broader perspective. Second, the study distinguishes dis-adoption decisions from adoption and never-adoption decisions and presents evidence of their differences. Third, the study identifies context-specific factors associated with household adoption decisions and discusses their policy implication in view of enhancing the sustained use of innovations at the smallholder level.

## 2. Methods and data

### 2.1 Study areas and sampling

We used data from the African Chicken Genetic Gain (ACGG) farm household survey collected in 2015/16 in Ethiopia, Tanzania, and Nigeria (Ethiopia: 20 October 2015–05 February 2016; Nigeria: September 02, 2015-November 5, 2015; Tanzania: August 25, 2015-October 19, 2015). In each country, the rural areas are represented by regional and sub-national zones. The survey was carried out to characterize existing smallholder chicken production systems and understand farmers’ preferences for chicken traits. The survey adopted multistage sampling approaches, including selecting regions, subnational zones, districts, villages, and households. Regions and subnational zones are selected purposively based on predefined criteria that include the population of chickens, number of smallholder chicken producers, the contribution of chicken to household income and diets, market share captured by smallholder producers, availability of feed for a growing chicken industry, and finally the diversity of agro-ecological zones. In each subnational zone, the districts were purposively selected, followed by selecting a group of villages in each district. The cluster of villages was selected purposely, and 6–18 villages were selected in each subnational zone. The number of villages selected per subnational zone was directly proportional to the human population of the subnational zone. A total of 63, 60, and 80 villages were selected in Ethiopia, Nigeria, and Tanzania, respectively. Finally, households in each village with at least two years of chicken-keeping experience were randomly selected. The total number of households selected in Ethiopia, Nigeria, and Tanzania was 1259, 1202, and 1202, respectively, or 3663 in total. The initial cleaning dropped 110 households due to missing values of one or more variables; therefore, we have an analyzable sample of 3553. Data collection involved using a structured questionnaire survey tool via face-to-face interviews facilitated by the Open Data Kit (ODK) tool. This study was approved by the Institutional Research Ethics Committee of the International Livestock Research Institute (ILRI IREC). The interview started with the presentation of a consent statement covering the study objectives, sampling methods, confidentiality, anonymity guarantees, and a request for confirmation of participation consent. In particular, all households sampled gave their written informed consent for inclusion before the interviews began, highlighting their informed agreement to participate in the study.

### 2.2 Data and empirical approaches

#### 2.2.1 Dependent variable

The dependent [decision] variable is derived from two indicators showing the current adoption status and previous adoption experiences of households that did not adopt improved chicken breeds. Improved chickens refer to chicken breeds developed to exhibit important and preferred traits such as higher egg and meat productivity, disease resistance, local adaptation, egg and meat taste preferred by consumers, etc. A combination of the two indicators resulted in a categorical variable with three levels i.e., ‘adopters’, ‘dis-adopters’, and ‘never-adopters’. ‘Adopters’ represent households that currently use improved chicken breeds; ’dis-adopters’ represent households that have adopted improved chicken breeds before but currently do not own any improved breed; ’never-adopters’ represent households that have never used improved chicken breeds. A summary of these indicators shows that the proportion of improved chicken adopters is higher in Ethiopia and lowest in Tanzania (Table 1). Compared to Ethiopia, the proportions of never-adopters and dis-adopters were higher in Tanzania and Nigeria. The overall dis-adoption rate was 18.7% in the three countries, which is greater than the overall adoption rate (16%).

**Table 1:** Improved chicken breeds adoption status by country.

Adoption status	Ethiopia	Nigeria	Tanzania	Total
Never-adopter	56.5	54.9	85.3	65.3
Dis-adopter	15.8	31.8	8.9	18.7
Adopter	27.7	13.4	5.8	16.0
Total	1,256	1,146	1,151	3,553

#### 2.2.2. Independent variables

We selected independent variables based on theoretical and empirical evidence from previous technology adoption studies. The variables include household characteristics (age and education level of household head), breed improvement practices (breed selection and culling), experience in using other improved livestock breeds, access to institutions (credit, training, and extension, and distance to main road), complimentary inputs (feed, vaccine and labor), household wealth (source of income, land size) and purpose of chicken production. We summarized these variables by type of adoption status (Table 2). Of the total sample households, 22.2% were female-headed. Only 10.7% of the sample households participated in chicken production and marketing-related training and extension services. Many more adopters participated in training and extension services than never-adopters or dis-adopters. Some producers had access to formal and informal loans. Formal loans refer to loans from banks, microfinance institutions, saving and credit groups, and non-governmental organizations. The informal loan refers to loans from informal lenders, friends, and relatives. The proportion of producers who took formal and informal loans was 24.4% and 14.3%, respectively. A higher proportion of adopters had access to formal loans than never-adopters and dis-adopters.

**Table 2:** Summary of categorical independent variables.

Variable	Category	Never adopter	Dis-adopter	Adopter	Total	Pearson Χ^2^
HH sex	Male	77.0	80.8	77.9	77.8	4.41
	Female	23.0	19.2	22.1	22.2	
Training	No	89.6	93.5	83.4	89.3	32.53***
	Yes	10.4	6.5	16.6	10.7	
Formal loan	No	79.5	72.9	62.7	75.6	72.06***
	Yes	20.5	27.1	37.3	24.4	
Informal Loan	No	86.0	84.0	86.4	85.7	1.97
	Yes	14.0	16.0	13.6	14.3	
Experience in ILB	No	97.2	94.5	90.0	95.5	55.95***
	Yes	2.8	5.5	10.0	4.5	
Prefer Improved breed	No	76.1	66.8	40.5	68.7	
	Yes	23.9	33.2	59.5	31.3	265.79***
Breed Selection	No	41.4	35.3	27.0	37.9	
	Yes	58.6	64.7	73.0	62.1	42.97***
Culling: Poor Egg Production	No	73.3	62.3	63.7	69.7	
	Yes	26.7	37.7	36.3	30.3	41.1***
Culling: Broodiness	No	90.1	83.3	90.7	88.9	
	Yes	9.9	16.7	9.3	11.1	26.72***
Purpose: Income generation	No	8.6	6.2	4.1	7.4	
	Yes	91.4	93.8	95.9	92.6	14.85***
Purpose: Consumption	No	9.9	13.5	18.8	12.0	
	Yes	90.1	86.5	81.3	88.0	34.72***
Sample size	N	2,292	657	560	3,509	

*Note*: *HH denotes household head*. *ILB denotes improved livestock breeds*.

A small proportion (4.5%) of the sample households have experience in adopting other improved livestock breeds. The proportion of producers who own other improved livestock breeds is higher for adopters than for others. Of the total respondents, 31.3% reported improved chicken as their primary preference, with adopters more interested in improved chicken than never-adopters. Sixty-two percent of the sample respondents practice breed selection, while 53% practice culling. Culling refers to the purposeful removal of chickens based on certain desired characteristics. Producers cull chickens mainly based on old age, low body weight, low egg production or productivity, poor egg quality, lack of broodiness, plumage color, disease concern, and low vitality. We included only low egg production or productivity (including poor egg quality), and lack of broodiness because of their considerable frequency. A higher proportion of dis-adopters practiced culling than adopters and never-adopters. Although most households raise chickens for income generation and own consumption, compared to never-adopters, a higher proportion of adopters and dis-adopters aim for income generation than own consumption.

Table 3 summarizes continuous independent variables. The literacy levels (in years of education) for dis-adopters and adopters are higher than for never-adopters. Dis-adopters are slightly older than adopters and never-adopters. Almost all the sampled households provide supplementary feeds and vaccines to chickens. However, relatively, adopters provide feeds for a higher number of months and administer vaccines more often than non-adopters and never-adopters. Total income source, a proxy for income diversification, is slightly higher for dis-adopters than for never-adopters and adopters. The income sources include other farm and non-farm activities, such as crop and livestock production and employment in non-agricultural sectors. The average time adopters spend on chicken management per week is higher than that of non-adopters and dis-adopters. The housing index, an indicator of the quality of chicken houses, shows adopters use better-quality houses than dis-adopters and never-adopters. Never-adopters own larger land sizes than adopters and dis-adopters. This may suggest that never-adopters may have a better opportunity in crop production activities. The Wald chi-square test for the equality of means, which allows the heterogeneity of continuous variables in the three groups, suggests a statistically significant association between independent variables and the adoption status of households, except for the age of the head.

**Table 3:** Summary of continuous independent variables.

Variable	Never-adopter (N = 2292)		Dis- adopter (N = 657)		Adopter (N = 560)		Pooled (N = 3509)		Wald chi^2^(2)
	Mean	SD	Mean	SD	Mean	SD	Mean	SD	
HH education	5.57	4.56	6.78	5.19	5.80	5.23	5.83	4.82	29.24***
HH age	48.43	14.03	49.70	13.57	48.45	14.47	48.67	14.02	4.61
Distance to road (in Km)	1.96	3.03	2.46	3.70	2.01	3.07	2.06	3.18	10.22***
Supplementary feed (months/year)	9.66	4.05	9.98	3.93	10.83	3.00	9.91	3.90	61.31***
No. Vaccination (year)	0.42	0.59	0.43	0.58	0.53	0.76	0.44	0.62	9.94***
Total Income sources	1.43	0.68	1.66	0.77	1.62	0.89	1.50	0.74	63.37***
Family labor (hr/week)	2.74	3.66	2.23	3.47	3.70	4.82	2.80	3.38	36.46***
Housing index	0.27	0.21	0.30	0.24	0.40	0.25	0.30	0.23	118.58***
Land Size (in Ha)	1.85	3.98	1.24	4.20	1.44	4.54	1.67	4.13	12.46***

Note: HH denotes household head, Km denotes kilometer, hr denotes hour, and Ha denotes hectare.

#### 2.3.3 Empirical approach

Researchers base their analysis of technology adoption decisions of farm households on different but related theoretical frameworks such as Expected Utility Theory (EUT), Random Utility Theory, Technology Acceptance Model (TAM), Theory of Reasoned Action (TRA), Theory of Planned Behavior (TPB), and the combination of different theories [21–24]. For example, the EUT suggests that improved technology adoption decisions are associated with greater expected utility than the utility obtained from traditional practices. According to this theory, farmers aim to maximize unobserved utility, and the probability of deciding to use a given technology should be equal to or greater than the utilities of all other alternatives. Other theories, such as TAM, associate the use of technology with perceived usefulness, perceived ease of use, and attitude toward use, and this theory is one of the most commonly used theories in adoption studies [22,24]. In general, theories in technology adoption can focus on predicting and explaining human behavior, the role of organizational characteristics, perceived outcomes, and beliefs about technology [22]. However, considering a combination of theories may be very helpful, given the limited perspective of individual theories, to fully explain the smallholder farmers’ adoption decisions [25,26]. We have also adopted this approach and considered various explanatory factors based on different theories and perspectives.

Technology adoption decisions are usually analyzed using discrete choice models. The most common models are binary logit and probit, Tobit, Heckman selection models, and other censored regression models [11,27–29]. The multivariate probit model has also been used in some cases to determine the adoption status of multiple technologies [30,31]. Most empirical studies consider adoption as a binary decision and rarely distinguish ‘never-adopters’ from ‘dis-adopters’. Only very few studies considered this distinction [13,32,33]. However, households who have never tried a technology and those who have had some experience using that technology before usually have different perspectives. Aggregating the two groups together in analyzing adoption decisions implies the implicit assumption that improved technologies are always superior and preferred by farmers than other alternatives, which may not usually be the case. Separating ‘never-adopters’ and ‘dis-adopters’ can have multiple benefits, such as identifying the most important factors that affect the initial adoption decision of technology and factors associated with the sustained use of the technologies [33].

Consequently, we apply the multinomial logit model (MNL), which extends binary logistic regression and caters to the three categories of our dependent variables [34]. The multinomial model fits the binary logit model for all possible alternatives the dependent variable can assume. Although multinomial logistic regression does not assume normality, linearity, or homoscedasticity, it assumes independence of the alternatives, and this shall be tested for model validity.

The multinomial logit model is formulated based on the random utility theory such that a household decides to use a technology or not based on the utility function that has deterministic [explainable] and stochastic [random] components [35]. For a household that adopts a given technology, the adoption happens if the perceived utility of adopting the technology is greater than otherwise. In our context, a farmer decides to adopt (A) an improved chicken breed if:

$$A{=1(U}_{Ad}-U_{NAd}={PB}_{i}^{*}>0),\;i=1,\ldots ..,n$$ (1)

where *U*_*Ad*_ refers to the perceived utility of adoption, *U*_*NAd*_ refers to the utility of never-adoption or dis-adoption, and ${PB}_{i}^{*}$ is the perceived benefit of adopting over otherwise. ${PB}_{i}^{*}$, the adoption of improved chicken breeds, is a latent variable explained by observed household, farm, and other characteristics and constitutes a random component that is not explained.

In specifying the multinomial model, we set the ‘never-adoption’ option as the base category. Let *P*_*ij*_ be the probability that an individual *i* makes the adoption decision *j*; i.e., *P*_*ij*_ = *P*(*individual i chooses adoption status j*) where *i* ranges from 1 to n, and j takes the values 0 [never-adopted], 1 [dis-adopted], and 2 [adopted]. Given **X**, 1xK vector of explanatory variables with 1 as its first element, and ***β***_***j***_ is Kx1 vector of estimated coefficients, *P*_*ij*_ can be computed in a multinomial logit as:

$$P_{ij}(y_{i}=j|X)=\frac{exp(X_{i}\beta_{j})}{1+\sum_{h=1}^{2}exp(X_{i}\beta_{h})}$$ (2)

For the baseline category (j = 0 = Never-adopter), this probability is given by:

$$P_{ij}(y_{i}=NAD|X)=\frac{1}{1+\sum_{h=1}^{2}exp(X_{i}\beta_{h})}$$ (3)

The *j* log-odds ratios can be computed as:

$$\ln\left(\frac{P_{ij}}{P_{ih}}\right)=X_{i}^{\prime }(\beta_{j}-\beta_{h})=X_{i}^{\prime }\beta_{j},\;if\;h=0.$$ (4)

The MNL model is best estimated with the maximum likelihood method [36]. Hence, for each *i*, the conditional log-likelihood can be written as:

$$L_{i}(\beta )=\sum_{j=0}^{J}1[y_{i}=j]ln[P_{j}(X_{i},\beta )]$$ (5)

## 3. Results and discussion

### 3.1 Test for indistinguishability of ‘never-adoption’ and ‘dis-adoption’

One of the significant limitations in existing technology adoption studies is merging ‘never-adopters’ and ‘dis-adopters’ into ‘non-adopters’, to proceed and compare them with adopters. Before estimating the MNL model, we tested if never-adopters are indistinguishable from dis-adopters. The Wald test indicates that all three categories are distinguishable with a significant level of 1% (Table 4). This suggests potential bias in parameter estimates if we combine never-adopters and dis-adopters to compare them with adopters in the adoption decisions model.

**Table 4:** Test for indistinguishability of dis-adopters and never-adopter.

Combinations	Χ^2^	DF	P> Χ^2^
Never-adopters and dis-adopters	383.904	22	0.000
Never-adopter and adopters	471.807	22	0.000
Dis-adopters and adopters	283.495	22	0.000

Note: Χ^2^ denotes chi-squared statistic, and DF denotes degrees of freedom.

### 3.2 Determinants of adoption of improved chicken breeds

We applied the MNL model to explore factors associated with improved chicken adoption and dis-adoption decisions, with never-adopters as a reference. The model was statistically significant (p <<< 0.001). Table 5 presents the estimated marginal effects based on the MNL model estimated on the pooled data. The estimated marginal effects demonstrate the effect of different independent variables on ‘never-adoption’, ‘dis-adoption’, and ‘adoption’ decisions. As indicated in the indistinguishability test above, never-adopters have different characteristics from adopters and dis-adopters.

**Table 5:** Marginal effects of estimated parameters from MNL-Pooled.

Variable	Never-adopter		Dis-adopter		Adopter	
	Coef.	SE	Coef.	SE	Coef.	SE
Head age (Years)	-0.001**	(0.001)	0.000	(0.000)	0.001***	(0.000)
Head Gender (Female)	-0.005	(0.018)	-0.026*	(0.015)	0.031**	(0.015)
Head Education (Years)	-0.011***	(0.002)	0.004***	(0.001)	0.007***	(0.001)
Training (Yes)	-0.017	(0.026)	-0.043**	(0.021)	0.060***	(0.021)
Distance to road (ln km)	0.003	(0.011)	0.018**	(0.009)	-0.021**	(0.009)
Income sources (Number)	-0.030***	(0.010)	0.025***	(0.008)	0.005	(0.007)
Formal Loan (Yes)	-0.079***	(0.018)	0.054***	(0.016)	0.024*	(0.013)
Informal Loan (Yes)	0.010	(0.021)	-0.004	(0.018)	-0.006	(0.016)
Land size (ln ha)	0.071***	(0.013)	-0.052***	(0.012)	-0.020*	(0.010)
Supplementary Feed (Months)	-0.001	(0.002)	-0.005***	(0.002)	0.006***	(0.002)
Vaccination (Rounds)	-0.069***	(0.013)	0.020*	(0.011)	0.049***	(0.009)
Family labour (ln hours)	-0.005	(0.011)	-0.049***	(0.010)	0.055***	(0.008)
Housing Index	-0.239***	(0.032)	-0.003	(0.028)	0.241***	(0.024)
Improved Lk. Breed (Yes)	-0.194***	(0.038)	0.119***	(0.038)	0.074**	(0.029)
Prefer Improved breed (Yes)	-0.126***	(0.024)	0.046**	(0.021)	0.080***	(0.018)
Practice breed selection (Yes)	-0.061***	(0.017)	0.052***	(0.014)	0.009	(0.014)
Culling: Poor egg production (yes)	-0.053***	(0.018)	0.070***	(0.016)	-0.017	(0.013)
Culling: Not broody (Yes)	-0.067***	(0.025)	0.074***	(0.023)	-0.007	(0.019)
Main Purpose: Income (Yes)	-0.103***	(0.026)	0.053**	(0.021)	0.049**	(0.021)
Main Purpose: Consumption (Yes)	0.028	(0.023)	-0.039*	(0.021)	0.011	(0.015)
Nigeria	-0.074**	(0.031)	0.222***	(0.025)	-0.148***	(0.026)
Tanzania	0.236***	(0.028)	-0.009	(0.019)	-0.226***	(0.023)
Observations	3,509		3,509		3,509	

Note: Coef. denotes estimated coefficient, and SE denotes standard error of the coefficient.

The age of the household head has contrasting effects on adoption decisions. An increase in age decreases the likelihood of never-adopting and increases the likelihood of adoption. This suggests that as farmers gain more experience in poultry production and marketing activities, their interest in new breeds increases. Household head’s education, training, and extension services significantly affect adoption decisions. An increase in education is associated with a higher likelihood of dis-adoption, while training and extension services are associated with a lower likelihood of dis-adoption. On average, an increase in years of schooling decreases the probability of never-adopting by 1.1% and increases dis-adoption (the probability of trying and quitting) by 0.4% while also increasing adoption by 0.7%. Households with access to training and extension services on chicken production and marketing have a 6.0% higher likelihood to adopt and a 4.3% lower likelihood to dis-adopt than others, indicating the significance of providing practical skills needed for chicken production to use specific technologies effectively. These relationships imply the key role of education, training, and extension services in adopting and sustaining technology use. The positive effect of education and training on adoption decisions could be associated with a better understanding of technologies, improved access to information, reduced risk aversion behavior, and improved attitude toward risk [37]. Other studies have also reported the positive and significant effect of education on dis-adoption decisions [38].

Considering male-headed households as a reference, the probability of dis-adoption for female-headed households decreases by 2.6% while the probability of adoption increases by 3.1%. Female-headed households tend to adopt an improved chicken breed more often than male-headed households and are more likely to continue using improved chicken breeds. Households near all-weathered roads have a higher likelihood of adoption and a lower likelihood of dis-adoption. A unit increase in the log of distance to all-weather roads increases the probability of dis-adoption by 1.8% and decreases the likelihood of adoption by 2.1%. This suggests that households with better road access may have better access to the input and output markets and other institutional services, increasing the likelihood of adopting improved chicken. Since most of the improved chicken breeds are sourced from commercial companies and are usually found in urban areas, households in rural areas may not be able to access these breeds and other complementary inputs. Farmers with access to better roads can also supply more output to bigger markets than others, which can increase their profitability. These findings are consistent with comparable studies on improved technology adoption [12].

Access to finance increases households’ likelihood to adopt technologies, as farmers can purchase improved technologies and other complementary inputs [9]. Indicators of access to financial services include exposure to loans from formal financial institutions (i.e., banks, microfinance, and saving and credit associations) and to loans from informal sources (i.e., informal lenders and friends or family members). Exposure to loans from formal sources decreases the probability of never adopting by 8.0%, increasing the likelihood of dis-adoption and adoption by 5.4% and 2.4%, respectively. This demonstrates the important role of access to financing in testing or the continued use of sustained adoption of improved chicken technology.

A strong and positive relationship between access to formal financing and dis-adoption decisions is expected. This is because households with access to formal finance have improved liquidity, which enables them to have better access to chicken production inputs and greater investment and risk mitigation capacity. The absence of a statistically significant association between informal loans and adoption decisions can be explained by the limited scale of the loan for production purposes and the diverse use of informal loans by households for non-agricultural activities compared to formal loans. For example, among formal loan takers, 71.3% used the loan for the purchase of inputs/equipment, livestock purchase & management, and running businesses, while 50.8% of informal loan users used the loan for similar purposes. However, while 20.1% of formal loan takers used the loan to pay school fees and purchase household items, 46.1% used it for similar purposes. Interestingly, a higher proportion of formal loan users used the loan for the purchase and management of livestock. Therefore, policy options for creating access to financial services may focus on developing innovative formal financial instruments tailored for improving chicken production activities.

The duration of supplementary feeding, total family labor used, and housing index are positively associated with adoption decisions and negatively associated with never-adoption and dis-adoption decisions. A unit increase in feeding months, hours of labor, and housing index increases the probability of adoption by 0.6%, 5.5%, and 24.1%, respectively. On the other hand, a unit increase in feeding months and hours of family labor decreases the likelihood of dis-adoption by 0.5% and 4.9%. This is expected as improved breed adoption demands supplementary feeds, and farmers with local breeds mostly use limited additional feeds. Access to vaccination services also increases the likelihood of adoption by 4.9% and decreases the likelihood of never-adoption by 7.0%. Positive and significant association of complementary inputs with adoption decisions is expected, as improved breeds need better feeding, vaccination, housing, and other management practices than local breeds. This suggests the need to avail a bundle of integrated technologies rather than individual components and build producers’ capacity for sustained adoption.

The model results also show that a unit increase in land size increases the probability of never-adoption by 7.1% and decreases dis-adoption and adoption by 5.2% and 2.0%, respectively. This is mainly due to the limited association between smallholder chicken production and land size [39], as there might not be significant direct fixed costs associated with land size and smallholder chicken production. Additionally, households with larger land sizes can prioritize crop production or other livestock activities, whereas those with smaller land sizes can view chicken production as a strategy for income diversification. Households with smaller land sizes can consider chicken production as an important component of their livelihood and invest more in improved chicken breeds. In our sample, dis-adopters and adopters of improved chicken breeds are more constrained by land size compared to never-adopters. Unlike other agricultural activities, chicken production may not require a large land size and can provide optimal yield with a smaller land size.

A unit increase in the number of income sources decreases the likelihood of never-adoption by 3.0% and increases the likelihood of dis-adoption by 2.5%. However, its effect on adoption decisions is not statistically significant. Having a diversified income source can have both positive and negative effects on the adoption of technology. The positive effect can be associated with risk mitigation, increased investment capacity, and better access to inputs and services [40,41]. Negative effects can be associated with the inability of households to effectively manage their time and resources, risk aversion, and competing priorities [42]. Farmers with diversified income may be more risk-averse and have other competing livelihood activities that make prioritizing investments in new technologies challenging.

The household’s experience with other improved livestock technologies is strongly correlated with the adoption of improved chicken breeds and is inversely related to the status of never-adoption. Owning improved livestock breeds increases the likelihood of improved chicken adoption by 7.4% and decreases the likelihood of never-adoption by 19.4%. Similarly, households that raise other improved livestock breeds are 11.9% more likely to attempt and discontinue improved chicken adoption. The strong association between the adoption of other improved livestock breeds and the adoption of improved chicken breeds can be attributed to the increased confidence and familiarity of farmers with improved technologies, the lessons learned from previous technology adoption, the allocation and use of well-developed farm resources and the improved access to networks and information. However, farmers who have faced failure or loss due to technology adoption may be more cautious and risk-averse when considering new technologies.

The preference of households for better chicken breeds, breed selection practices, and experience in culling are crucial factors in adoption and dis-adoption decisions of improved chicken breeds. Preference for improved chicken breeds may show farmers’ belief in possible outcomes of technologies. Households who prefer improved or exotic chicken breeds have 8.0% and 4.6% higher probability of adoption and dis-adoption decisions, respectively. In contrast, households who prefer improved chicken breeds have 12.6% less likelihood of never-adoption. This may suggest the role of positive expectations in the adoption and continued use of technologies. Farmers’ preferences can result from a combination of factors such as economic and market trends, social and cultural influences, and environmental concerns. A positive change in preference due to increasing demand or a better economic return often leads to the adoption of technologies.

Unlike preference for improved chicken breeds, the effect of breed selection and culling practices on adoption status is statistically insignificant. However, households that practice breed selection have a 5.2% higher probability of dis-adoption and a 6.1% lower probability of never-adoption. Farmers who actively participate in breed selection practices may be willing to try different breeds, seek information on available breeds, compare their performances, evaluate traits, and assess how well these breeds align with their production goals and practices. Smallholder farmers often consider several factors when selecting chicken breeds, including egg productivity, body size and growth rate, plumage color, taste of eggs and meat, resistance to disease, adaptability to the local environment and consumer preferences[43]. Preference and breed selection practices can indicate the interest and exposure of farmers in improved chicken breeds and their efforts to improve the production and productivity of their flocks, which can play a key role in adoption decisions.

Similarly, the status of adoption decision is positively and negatively associated with culling practices of households. Farmers remove unproductive or unhealthy chickens to improve the production and productivity of their flock. Households that practice culling may be more proactive in managing their flocks and more receptive to adopt new technologies and practices, as they prioritize productivity and efficiency, which are key drivers for adopting improved breeds.

Unlike others, poor egg quality, low production/productivity, and non-broodiness of the hen seem to be triggering factors for dis-adaptors to cull chickens. Households experienced poor chicken egg quality and egg productivity, and non-broody hens have 7.0%, and a 7.4% higher probability of dis-adoption than others. Dis-adopters seem to look for good quality and higher egg productivity and broody hen breeds than others. Households that look for egg quality, egg productivity, and broodiness have a 5.3% and 6.7% lower likelihood of never-adopting than others. The above indicators suggest that dis-adoption decisions are strongly associated with breed performance and trait preference of the household. A key insight from these results is the importance of understanding farmers’ preferences for chicken traits and breed selection practices to promote the adoption and continued use of improved chicken breeds. This may involve testing the performance of breeds under smallholder management conditions and understanding farmers’ preferences during technology generation and dissemination efforts.

Household production objectives are essential in chicken production and marketing decisions. Households raising chickens mainly aimed at income generation are more likely to adopt improved chicken breeds than households aiming for consumption. Households aiming for income generation have a 4.9% and 5.3% higher likelihood of improved chicken breed adoption and dis-adoption, respectively. These households have a 10.3% lower likelihood of never-adoption. Mostly, improved chicken breeds are perceived to increase production and productivity. Therefore, households that prioritize income generation may invest in technologies that generate better egg and meat productivity, reduce average production costs, or open new market opportunities. On the other hand, households that focus on raising chickens for their own consumption may have different considerations, such as better-quality products, meat and egg tastes and plumage color of chickens. These households may also use chickens for cultural and ceremonial purposes [43] and may not prioritize chicken characteristics like high egg and meat productivity to generate better income, resulting in less interest in adopting improved chicken breeds.

### 3.3 Comparative effect of independent variables on adoption status

Evidence on the effects of each independent variable on the three alternative outcomes provides additional intuitions about their relative importance for research, development, and policy interventions. The comparative effect analysis shows that some of the independent variables have heterogeneous effects on the three adoption decisions. Particularly, the effects of the independent variables are considerably different between dis-adopters and never-adopters (see Table 6). For example, higher household head education, access to formal loans, vaccination practices, income diversification, quality housing, other improved livestock breeds and breed selection experience, an inclination for improved chicken breeds, culling practices, and prioritizing income generation as main objective increase the likelihood of dis-adopting compared to that of never-adopting. On the other hand, higher feed supplementation, intensified use of family labor, larger land size holding, and prioritizing consumption as a production objective reduce the likelihood of dis-adopting compared to never-adopting. This evidence shows the presence of a clear distinction between dis-adopting and never-adopting decisions.

**Table 6:** Comparative effects of independent variables in each outcome.

Variable	Dis-adopter vs Never-adopter		Adopter vs Never-Adopter		Adopter vs Dis-adopter	
	b	%	b	%	b	%
Head age (Years)	0.003	0.3	0.012***	1.3	0.009*	0.9
Head Gender (Female)	-0.147	-13.6	0.241*	27.2	0.387**	47.3
Head Education (Years)	0.047***	4.8	0.80***	8.3	0.033**	3.4
Training (Yes)	-0.233	-20.8	0.441***	55.4	0.664***	96.2
Distance to road (ln km)	0.096	10.0	-0.169**	-151.5	-0.264***	-23.2
Income sources (Number)	0.215***	24.0	0.105	11.1	-0.110	-10.4
Formal Loan (Yes)	0.473***	60.6	0.358***	43.0	-0.116	-10.9
Informal Loan (Yes)	-0.047	-4.6	-0.066	-6.4	-0.019	-1.9
Land size (ln ha)	-0.459***	-36.8	-0.313***	-26.9	0.146	15.7
Supplementary Feed (Months)	-0.023*	-2.3	0.049***	5.0	0,072***	7.5
Vaccination (Rounds)	0.274**	31.5	0.532***	70.2	0.258**	29.5
Family labour (ln hours)	-0.271***	-23.8	0.432***	54.1	0.904***	102.2
Housing Index	0.530**	69.9	2.391***	992.2	1.861***	542.9
Improved Lk. Breed (Yes)	1.042***	183.5	0.976***	165.4	-0.066	-76.4
Prefer Improved breed (Yes)	0.530***	69.9	0.859***	136.1	0.329*	39.0
Practice breed selection (Yes)	0.489***	56.6	0.201	22.3	-0.247	-21.9
Culling: Poor egg production (yes)	0.501***	65.0	-0.017	-1.2	-0.512***	-40.1
Culling: Not broody (Yes)	0.530***	69.9	0.104	11.0	-0.426**	-34.7
Main Purpose: Income (Yes)	0.569***	76.6	0.654***	92.4	0.085	8.9
Main Purpose: Consumption (Yes)	-0.271*	-23.7	0.018	1.9	0.290	33.6
Nigeria	1.249***	248.7	-0.726***	-51.6	-1.975***	-86.1
Tanzania	-0.529***	-40.9	-2.345***	-90.4	-1.820***	-83.8

Note: b denotes raw coefficient, and % denotes percent change in odds for unit increase in X.

### 3.4 Major reasons for dis-adoption

The key reasons for the dis-adoption of improved chicken breeds can be broadly summarized into technology characteristics, lack of sustained supply of integrated technology packages, limited knowledge and technical capacity of producers, and inadequate access to markets and services.

#### 3.4.1 Technology characteristics

Our findings reinforce the importance of technology characteristics in influencing technology adoption and diffusion rates in smallholder production systems, in line with the findings of previous research [23]. The strong link between the adoption and dis-adoption of improved chicken breeds and the preference for improved breeds (exotic and crossbred) and experience with other improved livestock species production practices suggests the presence of common interests among adopters and dis-adopters. Interestingly, some dis-adopters express more interest in improved breeds than in local breeds despite not currently using them, indicating a perceived utility of improved breeds.

Compared to never-adopters, higher proportions of adopters and dis-adopters practice breed selection and culling based on specific breed characteristics such as egg yield, egg quality, broodiness, disease resistance, and body weight. This reflects the effort of households to identify breeds suitable for their production environment and, hence, their production goals. The likelihood of dis-adoption is usually higher for households that have experience with improved breeds with low profitability. This finding aligns with other studies that have found a positive association between low-quality products and higher dis-adoption rates of improved agricultural technologies [38], which highlights the importance of engaging farmers in technology generation and dissemination efforts.

#### 3.4.2 Lack of sustained supply of bundle of technologies

Improved chicken adopters and dis-adopters primarily engage in chicken production to generate additional income, indicating a commercial orientation not seen in never-adopters. This underscores the importance of ensuring adequate access to the input and output markets. Furthermore, the stronger and positive association between indicators related to input use, such as better housing, supplementary feed, vaccination, and family labor, and the decision ’adoption’ compared to decisions ’never-adoption’ and ‘dis-adoption’ supports this notion.

Furthermore, the positive association between dis-adoption and chicken culling practices based on broodiness, or the negative associations between this indicator and ‘never-adoption’, may suggest farmers’ interest in maintaining a steady supply of replacement flocks. A lack of a reliable supply of chicks is a significant constraint in improved chicken breed-based production systems in developing countries [9]. Therefore, establishing a sustained delivery system for a bundle of technologies, including improved chicken breeds or self-multiplying chickens suitable for smallholder management conditions, should be an integral part of innovations that aim to transform the traditional smallholder production system.

#### 3.4.3 Limited knowledge and technical capacity

Sustained adoption of improved agricultural technologies requires adequate knowledge and skills to integrate the new technologies into existing production practices. This is reflected in the observed strong association between adoption decisions and education of household heads and access to training and extension services. Although the positive association between education level and dis-adoption may seem unexpected, it can be explained by the dynamics of adoption decisions of households. Education enhances critical thinking skills essential for understanding and evaluating technologies, and farmers with better education may be in a better position to optimize technologies. Educated farmers can make better decisions about the continued use of technologies as they better understand the costs and benefits of technologies and are expected to have better risk management skills. Educated farmers may quickly decide to adopt technologies, but they can also quickly dis-adopt if they unfavorably evaluate the return from adopting technologies. Education improves access to information due to better literacy and numeracy among farmers and helps establish better social networks. Compared to never-adopting and dis-adopting decisions, education and access to training and extension services have stronger effect on adopting decisions.

Similarly, customized training and extension services on chicken production and marketing play an essential role in reducing dis-adoption decisions by equipping farmers with practical and hands-on skills to effectively use improved production and marketing practices. Beyond developing skills, training helps farmers see the values of adoption and can reduce the risks of failure. This suggests that the dis-adoption of technologies can be minimized by integrating capacity building and technical support services with other innovative technologies before and after disseminating them to farmers.

#### 3.4.4 Inadequate access to market and services

The prioritization of income generation as the main production objective by improved chicken adopters and dis-adopters implies the market orientation of the production system. This requires timely and reliable access to information, finance, and input and output markets. However, compared to adopters, dis-adopters are situated farther from all-weather roads, indicating that they are located further from market locations. Hence, distance to the road is positively associated with dis-adoption decisions, unlike adoption decisions. Living in remote areas may hinder farmers’ continued use of improved chicken breeds compared to living in areas close to the market. Improving smallholder access to information and better market opportunities through the construction of roads and marketing infrastructures, establishing product aggregation and processing centers, linking with buyers and introducing digital communication tools enhance the continued use of improved breeds.

## 4. Conclusion

This study highlights smallholder farmers’ improved chicken adoption decisions in the context of developing countries. We used household level data and fitted a multinomial logit model to explore improved chicken adoption decisions. Farmers’ adoption decisions are associated with multiple factors such as household education, training and extension, formal loans, vaccination services, income diversity, housing quality, feed use, production objectives, and experience with other improved livestock species.

We observed clear differences between dis-adoption and never-adoption decisions, highlighting the two decisions are triggered by different factors. Interestingly, some dis-adopters still show interest in improved chicken breeds over local ones, indicating a perceived value in these breeds despite their discontinuation of use.

Moreover, our study shows that adopters and dis-adopters are more commercially oriented than never-adopters, and primarily produce chickens to generate income. This points out the necessity of ensuring farmers’ adequate access to inputs, output markets, and other services through building roads and marketing infrastructure, promoting collective actions, establishing product aggregation and processing centers, facilitating market linkages, and introducing innovative market information systems. Similarly, capacity building plays an important role, as demonstrated by the positive association between the household head’s literacy and access to training and extension services, and adoption decisions.

Our research has a couple of important limitations that similar researchers might take into consideration. First, we are using a cross-sectional dataset and hence, the cause-and-effect relationship we presented needs to be looked at only from theoretical and contextual viewpoints. Second, because of our data generation process, we could not consider farmers’ reverse decisions. Farmers could adopt a breed, decide to dis-adopt, and then revert to adopting. This is an issue that future research needs to look at, preferably based on longitudinal data.

## Supporting information

S1 Appendix Marginal effects of estimated parameters from MNL-Ethiopia. (DOCX)

S2 Appendix Marginal effects of estimated parameters from MNL-Nigeria. (DOCX)

S3 Appendix Marginal effects of estimated parameters from MNL-Tanzania. (DOCX)

S4 Appendix Descriptive summary of the independent variable by country. (DOCX)
