# Supplementary material for: The adoption non-adoption dichotomy: Why do smallholder producers dis-adopt improved chicken breeds?
Source: PLoS One. 2024 Oct 31;19(10):e0310060. doi: 10.1371/journal.pone.0310060 (PMC11527278; doi:10.1371/journal.pone.0310060)
Supplement: S1 Appendix — (DOCX) [file pone.0310060.s001.docx]

**S1 Appendix: Marginal effects of estimated parameters from MNL-Ethiopia.**

| **Variable** | **Never-adopter** | | **Dis-adopter** | | **Adopter** | |
| --- | --- | --- | --- | --- | --- | --- |
|  | **Coef.** | **SE** | **Coef.** | **SE** | **Coef.** | **SE** |
| Head age (Years) | -0.001 | (0.001) | 0.000 | (0.001) | 0.001 | (0.001) |
| Head Gender (Female) | -0.036 | (0.035) | -0.004 | (0.026) | 0.040 | (0.032) |
| Head Education (Years) | -0.014*** | (0.004) | 0.006* | (0.003) | 0.008** | (0.004) |
| Training (Yes) | -0.095** | (0.043) | -0.036 | (0.030) | 0.131*** | (0.041) |
| Distance to road (ln km) | 0.069*** | (0.020) | 0.006 | (0.016) | -0.075*** | (0.019) |
| Income sources (Number) | -0.007 | (0.016) | 0.006 | (0.012) | 0.001 | (0.014) |
| Formal Loan (Yes) | -0.115*** | (0.030) | 0.104*** | (0.024) | 0.012 | (0.025) |
| Informal Loan (Yes) | -0.065* | (0.038) | 0.057* | (0.033) | 0.009 | (0.034) |
| Land size (ln ha) | 0.038 | (0.029) | -0.026 | (0.023) | -0.012 | (0.027) |
| Supplementary Feed (Months) | -0.012*** | (0.004) | -0.011*** | (0.003) | 0.023*** | (0.005) |
| Vaccination (Rounds) | -0.020 | (0.032) | 0.041* | (0.023) | -0.021 | (0.027) |
| Family labour (ln hours) | -0.041* | (0.022) | -0.053*** | (0.018) | 0.094*** | (0.018) |
| Housing Index | -0.433*** | (0.068) | -0.001 | (0.055) | 0.434*** | (0.055) |
| Improved Lk. Breed (Yes) | -0.165*** | (0.051) | 0.069 | (0.044) | 0.095** | (0.047) |
| Prefer Improved breed (Yes) | -0.109*** | (0.033) | -0.003 | (0.027) | 0.112*** | (0.028) |
| Practice breed selection (Yes) | -0.121*** | (0.038) | 0.023 | (0.030) | 0.098*** | (0.032) |
| Culling: Poor egg production (yes) | -0.004 | (0.030) | 0.031 | (0.024) | -0.028 | (0.026) |
| Culling: Not broody (Yes) | -0.094* | (0.051) | 0.094** | (0.047) | 0.001 | (0.043) |
| Main Purpose: Income (Yes) | -0.033 | (0.085) | -0.079 | (0.076) | 0.112* | (0.066) |
| Main Purpose: Consumption (Yes) | -0.027 | (0.033) | -0.013 | (0.026) | 0.040 | (0.027) |
| Observations | 1,227 |  | 1,227 |  | 1,227 |  |

Note: Coef. denotes estimated coefficient, and SE denotes standard error of the coefficient.
