# Supplementary material for: The adoption non-adoption dichotomy: Why do smallholder producers dis-adopt improved chicken breeds?
Source: PLoS One. 2024 Oct 31;19(10):e0310060. doi: 10.1371/journal.pone.0310060 (PMC11527278; doi:10.1371/journal.pone.0310060)
Supplement: S2 Appendix — (DOCX) [file pone.0310060.s002.docx]

**S2 Appendix: Marginal effects of estimated parameters from MNL-Nigeria.**

| **Variable** | **Never-adopter** | | **Dis-adopter** | | **Adopter** | |
| --- | --- | --- | --- | --- | --- | --- |
|  | **Coef.** | **SE** | **Coef.** | **SE** | **Coef.** | **SE** |
| Head age (Years) | -0.001 | (0.001) | -0.001 | (0.001) | 0.001** | (0.001) |
| Head Gender (Female) | 0.043 | (0.033) | -0.062* | (0.033) | 0.019 | (0.025) |
| Head Education (Years) | -0.012*** | (0.003) | 0.004 | (0.003) | 0.008*** | (0.002) |
| Training (Yes) | -0.186** | (0.094) | 0.033 | (0.091) | 0.153* | (0.080) |
| Distance to road (ln km) | -0.081*** | (0.019) | 0.059*** | (0.018) | 0.022* | (0.013) |
| Income sources (Number) | -0.092*** | (0.020) | 0.076*** | (0.019) | 0.016 | (0.013) |
| Formal Loan (Yes) | -0.049 | (0.042) | 0.019 | (0.041) | 0.030 | (0.028) |
| Informal Loan (Yes) | 0.083** | (0.036) | -0.060* | (0.034) | -0.022 | (0.024) |
| Land size (ln ha) | 0.056** | (0.023) | -0.034 | (0.023) | -0.022 | (0.017) |
| Supplementary Feed (Months) | 0.006 | (0.004) | -0.003 | (0.004) | -0.003 | (0.002) |
| Vaccination (Rounds) | -0.100*** | (0.023) | 0.026 | (0.022) | 0.074*** | (0.013) |
| Family labour (ln hours) | -0.074*** | (0.023) | 0.015 | (0.022) | 0.059*** | (0.013) |
| Housing Index | -0.163*** | (0.060) | -0.007 | (0.059) | 0.169*** | (0.037) |
| Improved Lk. Breed (Yes) | -0.550*** | (0.016) | -0.318*** | (0.014) | 0.868*** | (0.014) |
| Prefer Improved breed (Yes) | -0.170*** | (0.046) | 0.114** | (0.047) | 0.056 | (0.035) |
| Practice breed selection (Yes) | -0.060** | (0.029) | 0.089*** | (0.029) | -0.029 | (0.020) |
| Culling: Poor egg production (yes) | -0.101*** | (0.038) | 0.130*** | (0.038) | -0.030 | (0.025) |
| Culling: Not broody (Yes) | -0.056 | (0.049) | 0.044 | (0.048) | 0.011 | (0.036) |
| Main Purpose: Income (Yes) | -0.131*** | (0.039) | 0.095** | (0.038) | 0.036 | (0.025) |
| Main Purpose: Consumption (Yes) | 0.080 | (0.050) | -0.074 | (0.051) | -0.006 | (0.034) |
| Observations | 1,140 |  | 1,140 |  | 1,140 |  |

Note: Coef. denotes estimated coefficient, and SE denotes standard error of the coefficient.
