# Supplementary material for: The adoption non-adoption dichotomy: Why do smallholder producers dis-adopt improved chicken breeds?
Source: PLoS One. 2024 Oct 31;19(10):e0310060. doi: 10.1371/journal.pone.0310060 (PMC11527278; doi:10.1371/journal.pone.0310060)
Supplement: S3 Appendix — (DOCX) [file pone.0310060.s003.docx]

**S3 Appendix: Marginal effects of estimated parameters from MNL-Tanzania.**

| **Variable** | **Never-adopter** | | **Dis-adopter** | | **Adopter** | |
| --- | --- | --- | --- | --- | --- | --- |
|  | **Coef.** | **SE** | **Coef.** | **SE** | **Coef.** | **SE** |
| Head age (Years) | -0.000 | (0.001) | 0.000 | (0.001) | 0.000 | (0.000) |
| Head Gender (Female) | -0.015 | (0.025) | 0.006 | (0.020) | 0.009 | (0.017) |
| Head Education (Years) | -0.001 | (0.003) | -0.000 | (0.002) | 0.001 | (0.002) |
| Training (Yes) | 0.021 | (0.024) | -0.026 | (0.020) | 0.006 | (0.016) |
| Distance to road (ln km) | 0.053*** | (0.020) | -0.039** | (0.017) | -0.015 | (0.013) |
| Income sources (Number) | -0.032** | (0.016) | 0.020 | (0.013) | 0.012 | (0.010) |
| Formal Loan (Yes) | 0.009 | (0.023) | -0.012 | (0.019) | 0.003 | (0.016) |
| Informal Loan (Yes) | 0.017 | (0.031) | 0.007 | (0.027) | -0.024 | (0.018) |
| Land size (ln ha) | 0.051*** | (0.016) | -0.052*** | (0.014) | 0.001 | (0.009) |
| Supplementary Feed (Months) | -0.003 | (0.003) | -0.002 | (0.002) | 0.004** | (0.002) |
| Vaccination (Rounds) | -0.055*** | (0.015) | 0.021* | (0.013) | 0.034*** | (0.010) |
| Family labour (ln hours) | 0.064*** | (0.016) | -0.066*** | (0.015) | 0.002 | (0.009) |
| Housing Index | -0.131*** | (0.039) | 0.035 | (0.032) | 0.096*** | (0.025) |
| Improved Lk. Breed (Yes) | -0.286*** | (0.063) | 0.208*** | (0.060) | 0.077* | (0.040) |
| Prefer Improved breed (Yes) | -0.153** | (0.076) | 0.056 | (0.059) | 0.097* | (0.056) |
| Practice breed selection (Yes) | -0.032 | (0.022) | 0.023 | (0.018) | 0.009 | (0.015) |
| Culling: Poor egg production (yes) | -0.087*** | (0.026) | 0.083*** | (0.023) | 0.003 | (0.016) |
| Culling: Not broody (Yes) | -0.058* | (0.031) | 0.075*** | (0.028) | -0.017 | (0.017) |
| Main Purpose: Income (Yes) | 0.008 | (0.046) | -0.001 | (0.039) | -0.007 | (0.031) |
| Main Purpose: Consumption (Yes) | 0.088 | (0.060) | -0.064 | (0.049) | -0.024 | (0.046) |
| Observations | 1,142 |  | 1,142 |  | 1,142 |  |

Note: Coef. denotes estimated coefficient, and SE denotes standard error of the coefficient.
