# Supplementary material for: The adoption non-adoption dichotomy: Why do smallholder producers dis-adopt improved chicken breeds?
Source: PLoS One. 2024 Oct 31;19(10):e0310060. doi: 10.1371/journal.pone.0310060 (PMC11527278; doi:10.1371/journal.pone.0310060)
Supplement: S4 Appendix — (DOCX) [file pone.0310060.s004.docx]

**S4 Appendix: Descriptive summary of the independent variable by country.**

| **Variable** | **Value** | **Ethiopia** | | | | **Nigeria** | | | | **Tanzania** | | | |
| --- | --- | --- | --- | --- | --- | --- | --- | --- | --- | --- | --- | --- | --- |
|  |  | **NAD**  **(%)** | **DISA**  **(%)** | **AD**  **(%)** | **Total**  **(%)** | **NAD**  **(%)** | **DISA**  **(%)** | **AD**  **(%)** | **Total**  **(%)** | **NAD**  **(%)** | **DISA**  **(%)** | **AD**  **(%)** | **Total**  **(%)** |
| Gender of Head | Male | 80.2 | 80.4 | 79.2 | 80.0 | 68.9 | 81.5 | 74.8 | 73.7 | 79.9 | 79.2 | 77.6 | 79.7 |
|  | Female | 19.8 | 19.6 | 20.8 | 20.0 | 31.1 | 18.5 | 25.2 | 26.3 | 20.1 | 20.8 | 22.4 | 20.3 |
| Training | No | 91.8 | 90.2 | 82.5 | 88.9 | 98.7 | 97.2 | 93.4 | 97.5 | 82.1 | 86.1 | 65.7 | 81.5 |
|  | Yes | 8.2 | 9.8 | 17.5 | 11.1 | 1.3 | 2.8 | 6.6 | 2.5 | 17.9 | 13.9 | 34.3 | 18.5 |
| Formal Loan | No | 68.5 | 46.9 | 55.0 | 61.3 | 90.0 | 85.9 | 78.8 | 87.2 | 80.6 | 76.2 | 65.7 | 79.3 |
|  | Yes | 31.5 | 53.1 | 45.0 | 38.7 | 10.0 | 14.1 | 21.2 | 12.8 | 19.4 | 23.8 | 34.3 | 20.7 |
| Informal Loan | No | 83.8 | 80.4 | 85.4 | 83.7 | 81.0 | 84.3 | 85.4 | 82.6 | 90.9 | 90.1 | 94.0 | 91.0 |
|  | Yes | 16.2 | 19.6 | 14.6 | 16.3 | 19.0 | 15.7 | 14.6 | 17.4 | 9.1 | 9.9 | 6.0 | 9.0 |
| Improved LK. Breed | No | 94.8 | 89.7 | 87.1 | 91.9 | 100.0 | 100.0 | 99.3 | 99.9 | 97.0 | 84.2 | 83.6 | 95.1 |
|  | Yes | 5.2 | 10.3 | 12.9 | 8.1 | 0.0 | 0.0 | 0.7 | 0.1 | 3.0 | 15.8 | 16.4 | 4.9 |
| Prefer Improved Breed | No | 28.7 | 19.6 | 12.3 | 22.7 | 93.9 | 84.3 | 84.1 | 89.6 | 98.3 | 95.0 | 86.6 | 97.3 |
|  | Yes | 71.3 | 80.4 | 87.7 | 77.3 | 6.1 | 15.7 | 15.9 | 10.4 | 1.7 | 5.0 | 13.4 | 2.7 |
| Breed Selection | No | 20.3 | 11.3 | 8.8 | 15.6 | 64.4 | 50.0 | 66.9 | 60.2 | 41.5 | 28.7 | 29.9 | 39.7 |
|  | Yes | 79.7 | 88.7 | 91.2 | 84.4 | 35.6 | 50.0 | 33.1 | 39.8 | 58.5 | 71.3 | 70.1 | 60.3 |
| Culling: Poor Egg Production | No | 67.0 | 57.7 | 57.0 | 62.8 | 84.8 | 74.0 | 86.8 | 81.7 | 76.9 | 53.5 | 65.7 | 74.2 |
|  | Yes | 33.0 | 42.3 | 43.0 | 37.2 | 15.2 | 26.0 | 13.2 | 18.3 | 23.1 | 46.5 | 34.3 | 25.8 |
| Culling: Poor Egg Quality | No | 86.1 | 80.9 | 85.1 | 85.0 | 97.6 | 93.4 | 97.4 | 96.2 | 89.4 | 68.3 | 89.6 | 87.6 |
|  | Yes | 13.9 | 19.1 | 14.9 | 15.0 | 2.4 | 6.6 | 2.6 | 3.8 | 10.6 | 31.7 | 10.4 | 12.4 |
| Culling: Lack of Broodiness | No | 92.9 | 87.1 | 91.2 | 91.5 | 90.1 | 85.4 | 90.7 | 88.7 | 88.2 | 68.3 | 88.1 | 86.4 |
|  | Yes | 7.1 | 12.9 | 8.8 | 8.5 | 9.9 | 14.6 | 9.3 | 11.3 | 11.8 | 31.7 | 11.9 | 13.6 |
| Income generation | No | 2.7 | 3.6 | 1.5 | 2.5 | 20.1 | 8.3 | 9.3 | 14.9 | 5.3 | 4.0 | 6.0 | 5.3 |
|  | Yes | 97.3 | 96.4 | 98.5 | 97.5 | 79.9 | 91.7 | 90.7 | 85.1 | 94.7 | 96.0 | 94.0 | 94.7 |
| Consumption | No | 22.7 | 23.2 | 26.0 | 23.7 | 7.0 | 9.9 | 8.6 | 8.2 | 2.8 | 7.9 | 4.5 | 3.3 |
|  | Yes | 77.3 | 76.8 | 74.0 | 76.3 | 93.0 | 90.1 | 91.4 | 91.8 | 97.2 | 92.1 | 95.5 | 96.7 |
| Sample size | N | 691 | 194 | 342 | 1,227 | 627 | 362 | 151 | 1,140 | 974 | 101 | 67 | 1,142 |

**Note**: NAD denotes Never adopter, DISA denotes Dis-adopter, and AD denotes Adopter.
